# Intracellular Singlet Oxygen Generation Mediated by Upconversion Nanoparticles in RAW 264.7 Macrophages

**DOI:** 10.1021/acsomega.6c04049

**Published:** 2026-09-04

**Authors:** Amanda Annunciação Farhat, Bernardina Amorín Uscata, Magnus Ake Gidlund, Hiro Goto, Marisa Helena Gennari de Medeiros, Hermi Felinto Brito, Paolo Di Mascio

**Affiliations:** † Departamento de Química, Instituto de Química, 28133 Universidade de São Paulo, São Paulo, São Paulo BR-05508-000, Brasil; ‡ Instituto de Medicina Tropical de São Paulo, Faculdade de Medicina, 232203 Universidade de São Paulo, São Paulo, São Paulo BR- 05403-000, Brasil; § Instituto de Ciências Biológicas, Universidade de São Paulo, São Paulo, São Paulo BR-05508-000, Brasil; ∥ Departamento de Bioquímica, Instituto de Química, 153989 Universidade de São Paulo, São Paulo, São Paulo BR-05508-000, Brasil

## Abstract

Upconversion nanoparticles (UCNPs) capable of generating reactive oxygen species under near-infrared (NIR) excitation have emerged as promising platforms for biomedical applications, particularly photodynamic therapy and immune-related modulation. Here, we investigate the biological application of core–shell UCNPs functionalized with a photosensitizer for the intracellular generation of singlet oxygen (^1^O_2_). The nanoparticles exhibited reproducible morphology, phase purity, and strong upconversion emission intensity, enabling efficient activation under NIR excitation. Murine RAW 264.7 macrophages were employed as a model of innate immune cells to evaluate nanoparticle uptake, cytocompatibility, and light-triggered ^1^O_2_ production. Luminescence-based assays and microscopy analyses confirmed efficient cellular internalization and localized ^1^O_2_ generation upon low-energy NIR excitation, while cytotoxicity studies demonstrated low basal toxicity in the absence of irradiation. Upon photoactivation, a significant reduction in cell viability was observed, consistent with photodynamically induced oxidative stress. These results further demonstrate that UCNP-based nanoplatforms can function as effective intracellular generators of reactive oxygen species in phagocytic immune cells and highlight their potential for photodynamic therapy (PDT), redox modulation, and to study molecular interactions at the cell membrane level.

## Graphical abstract

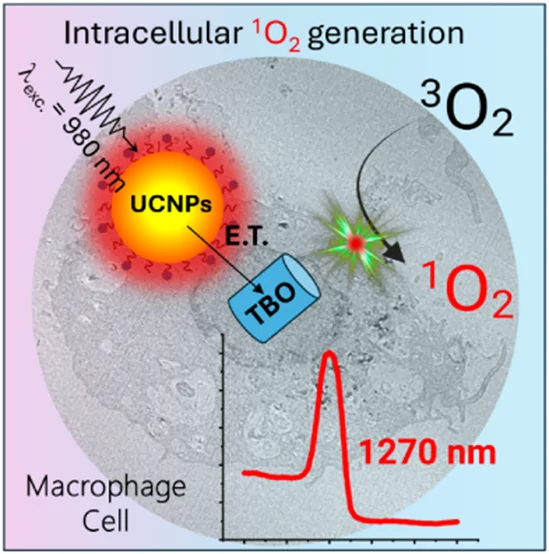

## 1 Introduction

Macrophages play a central role in the innate immune system and are among the first cell types to interact with foreign materials, including engineered nanomaterials. They are responsible for pathogen recognition, phagocytosis, antigen presentation, and modulation of inflammatory responses.^1–3^ The murine RAW 264.7 macrophage cell line is widely employed as an in vitro model to investigate macrophage–material interactions due to its high phagocytic activity, reproducibility, and ease of culture under controlled conditions.^4–6^ These features make RAW 264.7 cells particularly suitable for evaluating cellular uptake, intracellular trafficking, and biological responses to nanomaterials intended for biomedical applications.

Nanomaterials are predominantly internalized by macrophages through phagocytosis and endocytosis, processes that are strongly dependent on particle size, morphology, surface chemistry, and coating composition.^7–9^ Once internalized, nanomaterials can localize within endolysosomal compartments, where they may trigger oxidative stress, inflammatory signaling, or cell death pathways.^10,11^ Therefore, understanding how nanomaterial design influences macrophage responses is essential for both therapeutic applications and biosafety assessment.

Among the various nanoplatforms investigated for biomedical use, lanthanide-doped upconversion nanoparticles (UCNPs) have attracted considerable attention due to their unique optical properties. UCNPs convert near-infrared (NIR) excitation into visible or NIR emission via multiphoton processes, offering advantages such as deep tissue penetration, high photostability, minimal photobleaching, and low background autofluorescence.^12–15^ NaREF_4_-based UCNPs doped with rare-earth ions (RE^3+^) such as Er^3+^, Tm^3+^ and Yb^3+^ are among the most efficient systems, particularly under 980 nm excitation within the biological optical window.^16–18^ Among the available UCNP architectures, Er^3+^ /Tm^3+^ -based systems are particularly attractive for photodynamic applications because they exhibit intense red emission bands that overlap with the absorption region of phenothiazinium photosensitizers, including toluidine blue O (TBO), thereby favoring energy-transfer processes. In addition, these systems provide an alternative strategy to conventional Yb^3+^ -sensitized UCNPs while maintaining efficient upconversion luminescence and allowing flexible core@shell engineering.^18,19^

Beyond bioimaging, UCNPs have been explored as energy transducers in photodynamic systems by coupling them with photosensitizers capable of generating reactive oxygen species (ROS) upon excitation.^20–23^ In these systems, UCNPs absorb NIR light and emit photons at wavelengths overlapping with the absorption bands of photosensitizers, enabling the *in situ* generation of singlet oxygen (^1^O_2_). This strategy overcomes the limited tissue penetration of visible-light-activated photosensitizers and expands the applicability of photodynamic approaches in complex biological environments.^24–26^

Recent advances in UCNP-mediated photodynamic therapy have focused on the development of hybrid nanoplatforms capable of generating reactive oxygen species under NIR excitation through energy transfer to surface-bound photosensitizers. Several studies have demonstrated efficient singlet oxygen production using photosensitizers such as Rose Bengal, Eosin Y, and chlorin-based derivatives covalently attached or physically associated with UCNPs. These systems have shown improved photodynamic performance, enhanced tissue penetration, and increased therapeutic efficacy in vitro and in vivo, highlighting the importance of nanoparticle architecture, shell engineering, photosensitizer loading, and energy-transfer efficiency for ROS generation.^27–30^ Despite these advances, most studies rely on indirect methods to evaluate singlet oxygen formation, including chemical probes, fluorescent reporters, or biological end points. Direct observation of the characteristic 1270 nm phosphorescence emission of singlet oxygen in complex cellular environments remains comparatively uncommon due to the intrinsically weak emission signal and the optical challenges imposed by biological media. Consequently, studies providing direct evidence of intracellular singlet oxygen generation are still needed to strengthen the mechanistic understanding of UCNP-mediated photodynamic processes.

Singlet molecular oxygen is a highly reactive ROS that selectively oxidizes electron-rich biomolecules, including guanine in nucleic acids, unsaturated lipids, and specific amino acid residues in proteins, and its formation has been implicated in inflammation, photoaging, and carcinogenesis. Pioneering work by Di Mascio and coworkers has elucidated fundamental aspects of ^1^O_2_ generation and fate in biological and biomimetic systems, combining direct near-infrared detection of ^1^O_2_ phosphorescence with isotopically labeled endoperoxides and chemical traps to define molecular signatures of ^1^O_2_-mediated oxidation in lipids and DNA. More recently, this group has extended these concepts to complex systems, demonstrating ^1^O_2_ production in dark biological and oxidative processes as well as in photoinduced settings, and highlighting the roles of lipid hydroperoxides and other intermediates as endogenous sources of ^1^O_2_ in cells. These contributions provide a robust mechanistic framework for interpreting intracellular ^1^O_2_ generation and its downstream biological consequences in photodynamic and redox-active nanoplatforms.^31–33^

Despite significant progress in the development of UCNP-based photodynamic systems, important questions remain regarding the biological fate of these nanomaterials after cellular internalization. In particular, the relationship between nanoparticle architecture, surface functionalization, intracellular trafficking, and photodynamically induced biological responses requires further investigation. In immune cells such as macrophages, a comprehensive understanding of nanoparticle uptake mechanisms and intracellular localization is essential for the rational design of safer and more efficient photodynamic nanoplatforms.^34–36^

In this work, we report the application of core@shell NaErF_4_:Tm^3+^ @NaYF_4_ upconversion nanoparticles functionalized with poly­(maleic anhydride-*alt*-1-octadecene) (PMAO) and covalently linked to the photosensitizer toluidine blue O (TBO). Using RAW 264.7 macrophages as a cellular model, we investigate nanoparticle internalization by confocal and transmission electron microscopy, evaluate cytotoxicity under dark conditions, and demonstrate light-triggered intracellular singlet oxygen generation upon 980 nm laser irradiation. These results further highlight the potential of UCNP-based nanoplatforms for controlled photodynamic activity in immune phagocytic cells, with implications for photodynamic therapy, redox biology, and nanomedicine.

## 2 Experimental Section

### 2.1 Materials and Methods

#### 2.1.1 Materials

Oleic acid, 1-octadecene, sodium hydroxide, ammonium fluoride, erbium acetate, yttrium acetate, thulium acetate, poly­(maleic anhydride-*alt*-1-octadecene) (PMAO, *M̅w* ≈ 40 000 g mol^–1^), toluidine blue O (TBO), polyethylene glycol (PEG), chloroform (HPLC grade), acetone, ethanol, cyclohexane, borate buffer (pH 8.6), and HEPES buffer (50 mM, pH 7.5) were purchased from Sigma-Aldrich.

RAW 264.7 murine macrophages were obtained from Sigma-Aldrich. RPMI-1640 medium, fetal bovine serum (FBS), l-glutamine, penicillin, gentamicin, phosphate-buffered saline (PBS), dimethyl sulfoxide (DMSO), trypan blue solution, and DAPI were obtained from Thermo Fisher Scientific unless otherwise stated.

#### 2.1.2 Preparation of UCNP@PMAO@TBO

Hexagonal-phase β-NaErF_4_:Tm^3+^ core nanoparticles were prepared by a coprecipitation approach, following previously reported methodologies with minor adaptations.^37–39^ The Tm^3+^ concentration was fixed at 2 mol % relative to Er^3+^, based on emission optimization studies. The core@shell nanoparticles were synthesized using an optimized layer-by-layer epitaxial growth procedure designed to achieve controlled shell thickness while minimizing secondary nucleation. Further details regarding the optimization strategy are described in the associated patent application.^40^

Surface modification was achieved using PMAO at a surface density of approximately 1 polymer nm^–2^. Briefly, PMAO was dissolved in acetone, followed by addition of chloroform and UCNP dispersion. After solvent evaporation under nitrogen flow, dry UCNP@PMAO powders were obtained.

Covalent conjugation of toluidine blue O was performed through amide bond formation between PMAO anhydride groups and the amine functionality of TBO. The reaction was carried out in borate buffer (pH 8.6) in the presence of PEG, using two different anhydride-to-amine molar ratios (1:100 and 1:10). Toluidine blue O was incorporated as a minor fraction of the available amine-reactive sites, corresponding to approximately 0.5% of the total amine groups, while the remaining sites (∼99.5%) were occupied by PEG chains. This strategy was adopted to minimize dark toxicity while preserving efficient singlet oxygen generation under NIR excitation. The resulting UCNP@PMAO@TBO systems were purified by dialysis (100 kDa cutoff) and resuspended in HEPES buffer (pH 7.5). Additional experimental details related to UCNP synthesis and surface functionalization are provided in the (Supporting Information Section S1).

#### 2.1.3 Culture Cell

RAW 264.7 macrophages were cryopreserved in RPMI-1640 medium supplemented with 10% DMSO and 90% FBS. For experiments, cells were cultured in complete RPMI medium (RPMI-c) containing 10% FBS, 2 mM l-glutamine, 100 IU mL^–1^ penicillin, and 10 μg mL^–1^ gentamicin at 37 °C under 5% CO_2_ in a humidified incubator.

Cells were maintained in the exponential growth phase and subcultured according to confluence to ensure consistent morphology and viability.

#### 2.1.4 Experimental Groups

Cells were incubated with two UCNP core@shell sizes (UCNP I and UCNP II) and corresponding UCNP@PMAO@TBO formulations at a nanoparticle concentration of 100 μg mL^–1^. Two surface photosensitizer densities were evaluated (1 anhydride/100 amines and 1 anhydride/10 amines). DAPI was used for nuclear staining. A complete description of experimental groups is provided in the (Supporting Information Section S2).

#### 2.1.5 Cell Viability Assay

Cell viability was evaluated using the trypan blue exclusion assay. After incubation with nanoparticles for predefined time intervals (30 min to 24 h), cells were harvested, stained with trypan blue, and counted using a Neubauer hemocytometer. Viability was expressed as the percentage of unstained (viable) cells relative to the total cell count.

#### 2.1.6 Phagocytosis Inhibition Assay

To assess nanoparticle internalization, a low-temperature phagocytosis inhibition assay was performed. Cells seeded on glass coverslips were incubated with UCNP I@PMAO@TBO (100 μg mL^–1^) under two conditions: 37 °C (control) and 4 °C (ice). After 2 h, cells were washed, fixed, and analyzed by confocal microscopy.

#### 2.1.7 Phototoxicity Under NIR Irradiation

For phototoxicity evaluation, cells incubated with UCNP@PMAO@TBO were irradiated using a continuous-wavelength 980 nm laser (800 mW) for 60 s. Following irradiation, cells were harvested, and viability was assessed by trypan blue exclusion as described above.

#### 2.1.8 Characterization and Imaging Techniques

Structural and morphological characterization of UCNPs was performed by X-ray diffraction (XRD), transmission electron microscopy (TEM), and Dynamic Light Scattering (DLS). Optical properties and upconversion emission were analyzed by photoluminescence spectroscopy under 980 nm excitation. Cellular uptake and intracellular localization of UCNPs were evaluated by confocal laser scanning microscopy (CLSM) and TEM. Instrumental parameters and sample preparation procedures are described in the Supporting Information (Section S3.

## 3 Results and Discussion

### 3.1 Characterization of Prepared Nanomaterials

Two UCNP batches were prepared using the same β-NaErF_4_:Tm^3+^@NaYF_4_ core@shell architecture, dopant concentrations, surface modification protocol, and photosensitizer conjugation strategy. The only modification between the two formulations was a slight difference in the particle diameter, hereafter referred to as UCNP I and UCNP II.

Because the amount of PMAO added during surface modification was adjusted according to the nanoparticle surface area, the two batches required different amounts of polymer and consequently different amounts of TBO during functionalization. These differences reflect the distinct surface areas of the nanoparticles and do not imply a quantitative determination of the amount of TBO effectively bound to the surface.

The core@shell nanoparticles were characterized by transmission electron microscopy (TEM) to evaluate morphology, size distribution, and sample homogeneity. As shown in Figure 1, the particles exhibit uniform morphology and narrow size dispersion, with average diameters consistent with the expected values for the adopted synthetic protocol. The average particle diameter increased from 10.15 ± 0.48 nm for the UCNP I-core nanoparticles to 17.75 ± 1.69 nm after shell growth, considering the TEM image analysis by ImageJ software. Similar results were obtained for UCNP II, and the corresponding TEM images and size-distribution histograms are provided in Figure S1.

**Figure 1:**
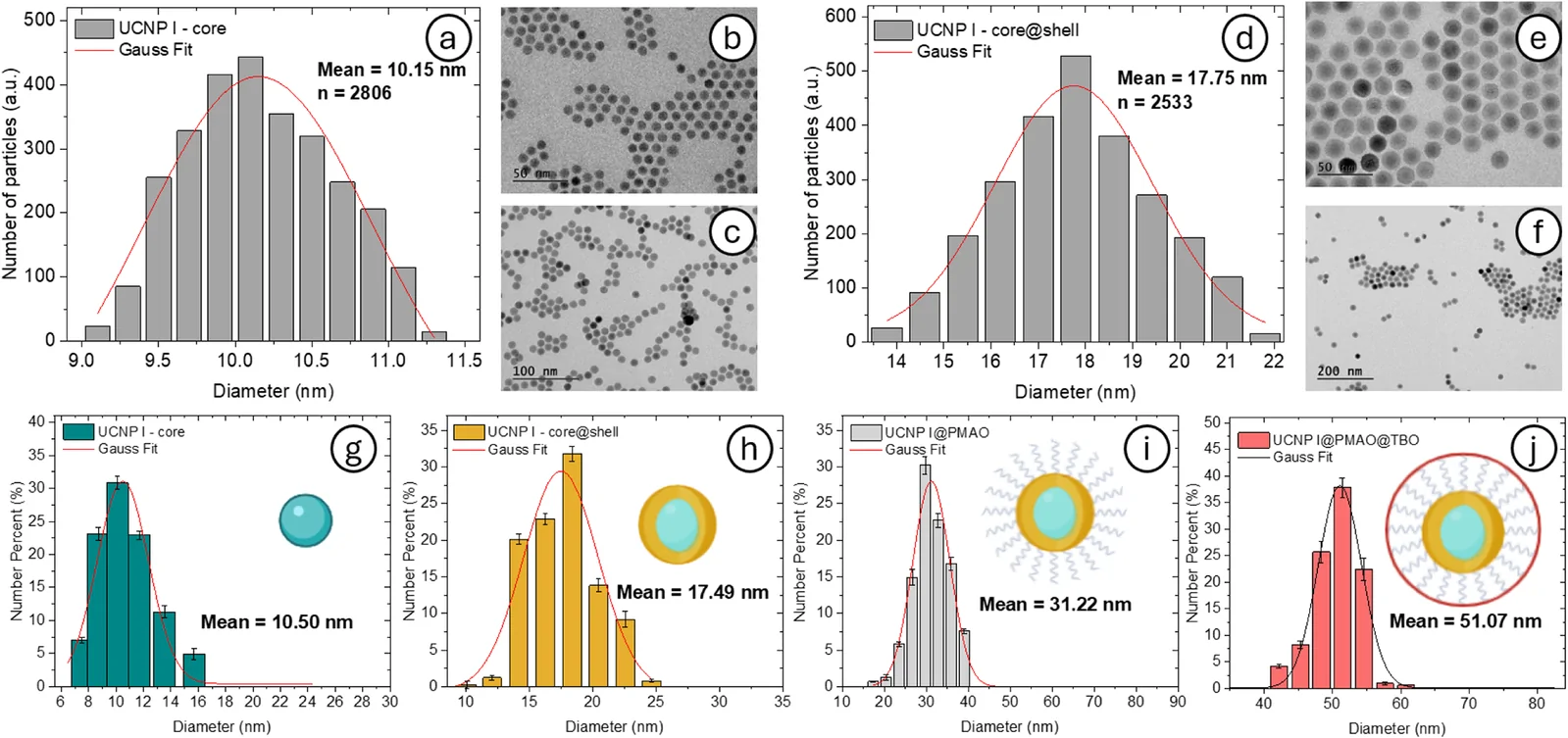
Structural and colloidal characterization of UCNP I. (a) size-distribution histogram of β-NaErF_4_^3+^ core nanoparticles obtained from TEM analysis (*n* = 2806 particles). (b,c) representative TEM micrographs of the core nanoparticles. (d) Size-distribution histogram of β-NaErF_4_^3+^@β-NaYF_4_ core@shell nanoparticles obtained from TEM analysis (*n* = 2533 particles). (e,f) representative TEM micrographs of the core@shell nanoparticles. Hydrodynamic diameter distribution obtained by dynamic light scattering (DLS) for nanoparticles (g) UCNP I core. (h) UCNP I core@shell. (i) UCNP I@PMAO after surface modification. (j) UCNP I@PMAO@TBO after photosensitizer conjugation.

To further assess shell growth, TEM analyses were performed both before and after the epitaxial coating step. The β-NaErF_4_:Tm^3+^ core nanoparticles exhibited a smaller average diameter (Figure 1a–c), whereas the final β-NaErF_4_:Tm^3+^@β-NaYF_4_ nanoparticles displayed a systematic increase in particle size while preserving morphology, monodispersity (Figure 1d–1f), and the characteristic hexagonal shape. The absence of a significant population of smaller particles in the final sample indicates that the observed size increase is associated with shell growth rather than the formation of a secondary nanoparticle population. Although conventional TEM does not allow direct visualization of the interface between the NaErF_4_:Tm^3+^ core and the NaYF_4_ shell due to their similar crystal structures and electron contrast, the increase in average particle diameter after the coating procedure is consistent with successful epitaxial shell deposition.

The analysis of the hydrodynamic radius of the core and core@shell by DLS also resulted in an average size corresponding to the size already analyzed (Figure 1g and 1h), in addition to corroborating the analysis of the homogeneity of the samples. Figure 1i shows the hydrodynamic radius for the UCNP@PMAO sample, which indicates an modification in the nanoparticle and Figure 1j shows the hydrodynamic radius of the functionalized nanomaterial (UCNP I@PMAO@TB), in which it is possible to verify an increase in the average size of the particles, without the presence of different particle populations. For the calculation of the amount of polymer and, consequently, of photosensitizer, the average size used for the core@shell was that obtained by TEM images.

Additional characterization by DLS, UV–Vis absorption spectroscopy, and X-ray diffraction is presented in Supporting Information Figures S1–S3 to compare the structural and surface properties of UCNP I and UCNP II before and after functionalization. In addition to morphological characterization, the optical properties of the nanoparticles were investigated to evaluate their suitability as NIR-activated photodynamic platforms. Efficient energy transfer between the UCNP and the photosensitizer requires spectral overlap between the upconversion emission and the absorption bands of the acceptor molecule. Figure 2a displays a broad absorption band between 500 and 700 nm of the toluidine blue TBO in water (blue line) as well as it exhibits the narrow emission bands at 490, 658, and 800 nm assigned to the ^4^S_3/2_→^4^I_15/2_, ^4^F_9/2_→^4^I_15/2_, and ^4^I_9/2_→^4^I_15/2_ transitions from the Er^3+^ ion (red line), respectively, showing a main emission band centered at 658 nm of the Er^3+^ ion. The significant spectral overlap in this region supports the feasibility of resonance energy transfer from the UCNP to the photosensitizer.

**Figure 2:**
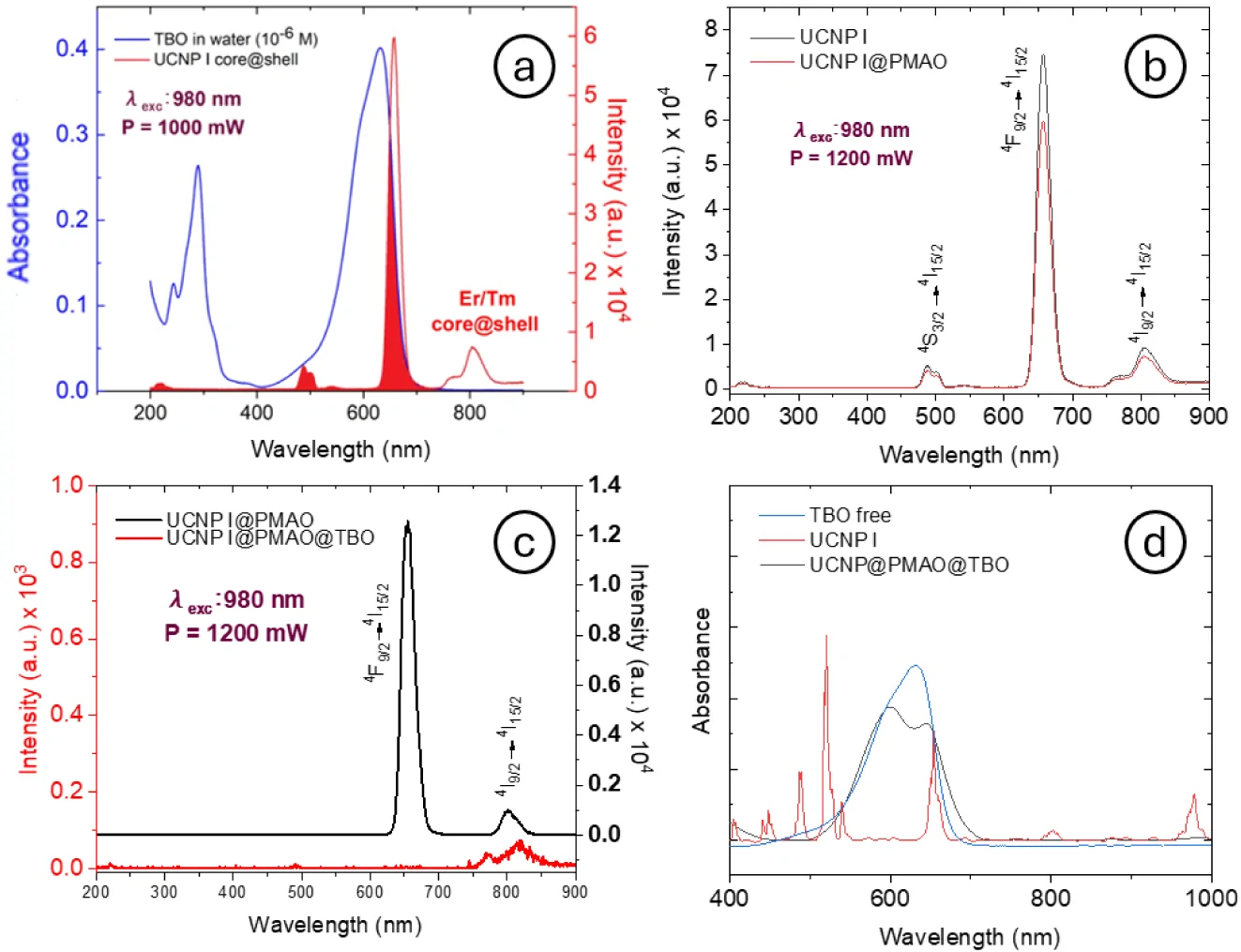
Optical characterization of UCNP I before and after surface functionalization. (a) spectral overlap between the emission spectrum of β-NaErF_4_:Tm^3+^ @β-NaYF_4_ nanoparticles dispersed in cyclohexane (λ_exc_ = 980 nm, *P* = 1000 mW) and the absorption spectrum of toluidine blue O (TBO) in water, demonstrating overlap in the 500–700 nm region. (b) Emission spectra of core@shell and core@shell@PMAO nanoparticles recorded in cyclohexane (λ_exc_ = 980 nm, *P* = 1200 mW). (c) Emission spectra of core@shell@PMAO nanoparticles recorded in water before and after TBO conjugation (λ_exc_ = 980 nm, *P* = 1200 mW), showing suppression of the 658 nm emission band. (d) UV–Vis absorption spectra of free TBO, core@shell nanoparticles, and UCNP@PMAO@TBO.

The luminescence properties of the nanoparticles before and after surface modification are presented in Figure 2b. The emission profiles of core@shell and core@shell@PMAO nanoparticles remain essentially unchanged, indicating that polymer coating does not significantly affect the upconversion process. To further verify system functionality, the emission spectra of core@shell@PMAO nanoparticles were recorded in the absence and presence of TBO (Figure 2c). The narrow emission bands at 490 and 658 nm of the Er^3+^ ion are observed for nanoparticles without a photosensitizer (Figure 2c, black line). Thus, their emission bands are significantly quenched after TBO functionalization (Figure 2c, red line), indicating efficient energy transfer from the UCNP nanomaterials to the photosensitizer.

Additional confirmation of TBO incorporation was obtained from the absorption spectra shown in Figure 2d. The unmodified UCNPs exhibit the characteristic narrow absorption bands associated with Er^3+^ transitions, whereas UCNP@PMAO@TBO displays an additional broad absorption band in the 550–700 nm region, corresponding to toluidine blue O. The preservation of the characteristic TBO absorption profile after surface functionalization indicates that the photosensitizer retains its optical properties upon incorporation into the polymer-coated nanoparticles. A moderate broadening of the absorption band was observed compared with free TBO, which is consistent with interactions between the dye molecules and the nanoparticle surface environment. Importantly, the absorption band remains spectrally overlapped with the red emission of Er^3+^ centered at approximately 658 nm, supporting efficient energy transfer from the UCNPs to the photosensitizer.

**Figure 3:**
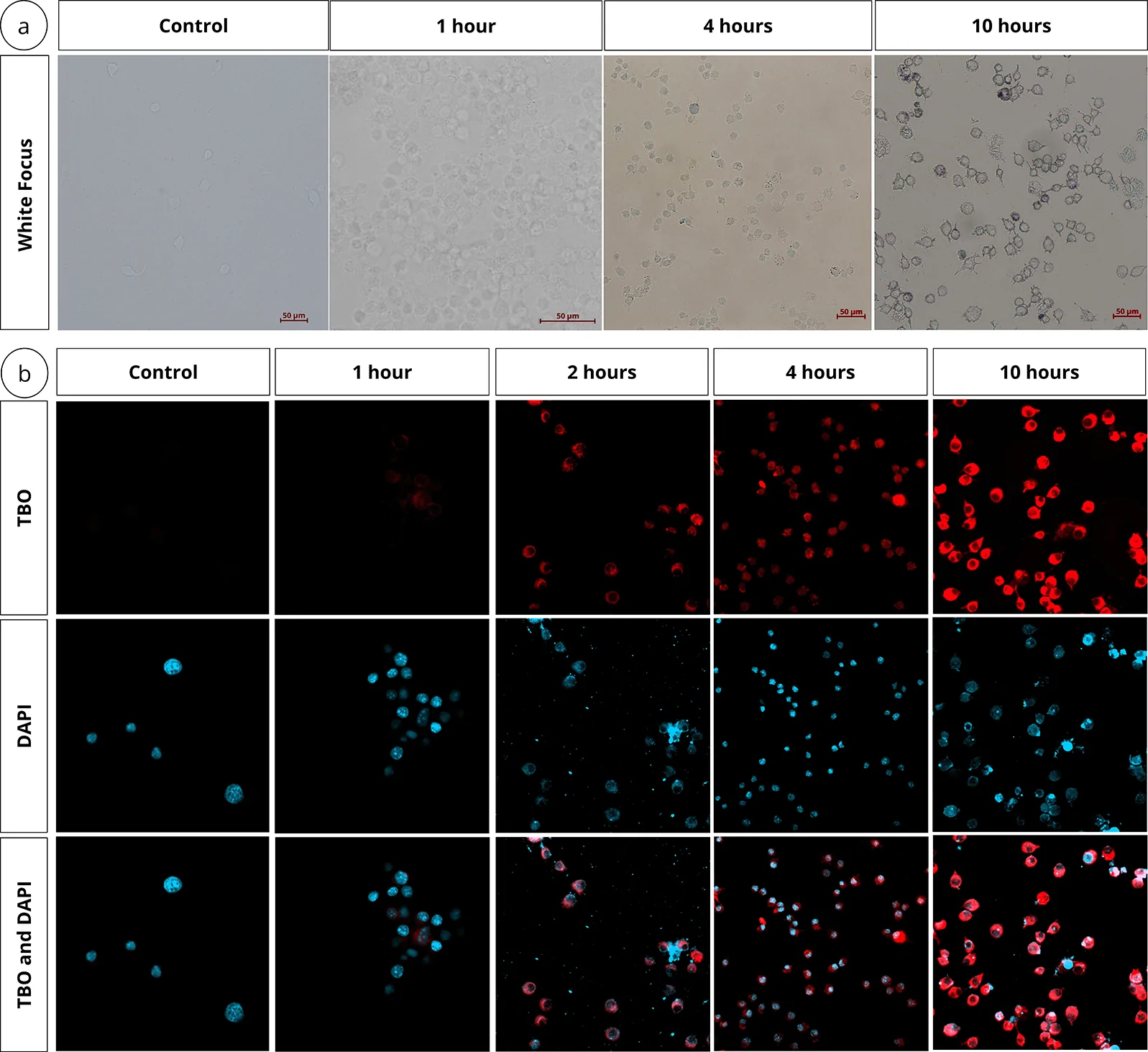
Confocal microscope macrophage images. (a) white focus (WF) microscopy images of control cells and cells incubated with UCNP@PMAO@TBO for 1, 4, and 10 h, showing preserved cellular morphology over time. (b) Confocal microscopy images of control cells and cells incubated with UCNP@PMAO@TBO for 1, 2, 4, and 10 h, acquired toluidine blue O (TBO), and 4′,6-diamidino-2-phenylindole (DAPI) channels, along with merged TBO/DAPI images.

### 3.2 Assay of Internalization and Colocalization of Nanomaterials in RAW 264.7 Macrophages

Slides containing cells incubated in the presence or absence of UCNP I@PMAO@TBO (Group I-1; TBO grafting ratio = 1 anhydride/100 amines; surface composition = 0.5% TBO and 99.5% PEG) were analyzed by confocal laser scanning microscopy using a 40× objective. Two fluorescence channels were used as references: DAPI was used to label cell nuclei (excitation at 465 nm and detection between 410–590 nm), and Alexa Fluor 610 (AF610), with excitation at 612 nm and detection between 585–700 nm, whose excitation and emission windows overlap with the absorption and emission bands of toluidine blue O (TBO).

Prior to biological assays, free (noncovalently bound) TBO was removed from the nanoparticle suspension by centrifugation, precipitation of the UCNPs, and complete discard of the supernatant. Additionally, after incubation, cells were thoroughly washed with PBS while still adhered to the substrates, following the same washing protocol for all experimental groups. Therefore, any AF610/TBO-related signal detected in the confocal images can be attributed exclusively to TBO molecules covalently anchored to UCNPs that were internalized by the cells.

All confocal images were acquired using identical acquisition parameters (laser power, gain, pinhole, and detector settings) for all samples to allow qualitative comparisons of fluorescence distribution.

Figure 3a presents representative images obtained in the white light (WF) channel for control cells and cells incubated with UCNPs for 1, 4, and 10 h. Across all incubation times, no significant changes in overall cell morphology were observed, indicating that exposure to UCNPs under the tested conditions does not induce detectable morphological damage. A gradual change in cell coloration is visible at longer incubation times, consistent with the accumulation of nanoparticle-loaded cells rather than extracellular staining or dye adsorption. Figure 3b compiles confocal images of control cells and cells incubated with UCNPs for 1, 2, 4, and 10 h, showing the DAPI, and TBO (AF610) channels, as well as their overlays, the images for all the times of incubation are available in the Supporting Information, in Figure S4. A detectable TBO-associated fluorescence signal is already observed after 2 h of incubation, indicating early internalization of UCNPs. From 8 h onward, a visually evident increase in AF610 signal intensity is evident, suggesting a time-dependent accumulation of UCNPs inside the cells. Analysis of the fluorescence overlays (Figure 3b, RBO and DAPI channels) reveals that the TBO signal does not spatially coincide with the DAPI-stained nuclei, even in a longer incubation time (10 h). Instead, the TBO fluorescence is predominantly localized in the perinuclear and cytoplasmic regions, close to the cell membrane. This spatial distribution indicates that UCNPs are internalized into the cytoplasm but do not translocate to the nucleus. Such localization is consistent with uptake via endocytic or phagocytic pathways and with the absence of nuclear-targeting moieties on the UCNP surface, as the photosensitizer is covalently attached to the outer polymer layer without specific nuclear-directing groups. Overall, the confocal microscopy results demonstrate efficient internalization of UCNP I@PMAO@TBO, confirm the intracellular (non-nuclear) localization of the nanoparticles, and validate that the observed fluorescence signal originates exclusively from nanoparticle-bound TBO rather than free dye or extracellular residues.

The biological samples prepared for ultrathin sectioning were analyzed by transmission electron microscopy (TEM). The images reveal the presence of electron-dense nanostructures within RAW 264.7 macrophages, predominantly localized in vesicular compartments compatible with phagosomes, as highlighted in Figure 4a.

**Figure 4:**
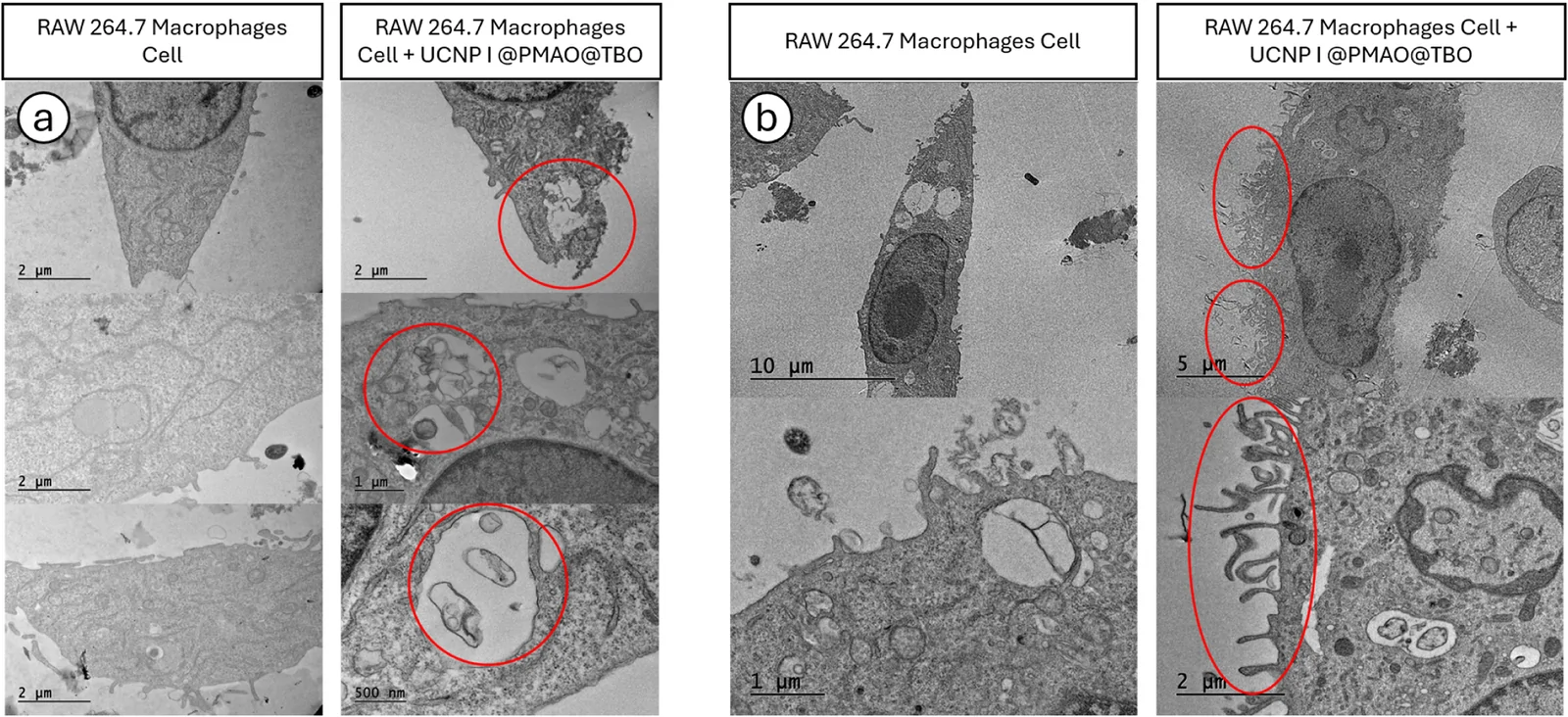
Transmission electron microscopy (TEM) images of RAW 264.7 macrophages. (a) control cells and cells treated with UCNP I@PMAO@TBO, highlighting vesicular structures compatible with phagosomes containing electron-dense nanomaterials (circled regions). (b) TEM images of control and UCNP-treated cells highlighting plasma membrane projections (pseudopodia), which are more abundant in the presence of UCNP.

These observations corroborate the confocal microscopy results, which indicated intracellular localization of UCNP without nuclear accumulation. In addition, TEM analysis allowed the observation of ultrastructural changes that could not be resolved by confocal microscopy.

Macrophages exposed to UCNP I@PMAO@TBO exhibited an increased number of plasma membrane projections compared to control cells, consistent with enhanced phagocytic activity. Such membrane protrusions, commonly referred to as pseudopodia, are commonly associated with macrophage interactions with foreign particulate materials and phagocytic activity. As shown in Figure 4b, cells treated with UCNP display more pronounced and abundant pseudopodial structures than untreated controls.

This behavior is in agreement with previous reports indicating that nanoscale materials can stimulate cytoskeletal reorganization and promote phagocytic uptake through direct interactions with the cell membrane and potential modulation of innate immune pathways. Together, these results support the hypothesis that UCNP internalization occurs predominantly via phagocytosis, with nanomaterials persisting within the cytoplasm, either enclosed in phagosomes or freely distributed in the cytosolic space.

To further confirm that UCNP internalization occurs predominantly via phagocytosis and to verify that the fluorescence detected in the TBO channel originates from the photosensitizer covalently bound to the nanoparticles, a temperature-dependent phagocytosis inhibition assay was performed.

Macrophages were seeded onto sterilized glass coverslips placed in 100 mm Petri dishes (3.2 × 10^5^ cells per dish, 10 mL of RPMI-c medium) and allowed to adhere. Subsequently, UCNP I@PMAO@TBO nanoparticles were added simultaneously to both experimental groups at the same concentration (100 μg mL^–1^). One group was maintained on ice (4 °C) to inhibit energy-dependent endocytic processes, while the other group was incubated at 37 °C in a humidified atmosphere containing 5% CO_2_ for 2 h, conditions known to favor active phagocytosis.

After incubation, the coverslips were thoroughly washed with PBS, mounted on glass slides, and analyzed by confocal microscopy. As shown in Figure 5, DAPI fluorescence is observed in both samples, confirming the presence of cell nuclei. However, no fluorescence signal is detected in the TBO channel for cells maintained at 4 °C, whereas a clear intracellular TBO signal is observed in cells incubated at 37 °C. These results indicate that nanoparticle internalization is strongly suppressed under low-temperature conditions, confirming the predominance of energy-dependent uptake processes. Combined with the TEM observations, which revealed membrane protrusions surrounding nanoparticle aggregates and their localization within intracellular vesicles, these findings support phagocytosis as a major uptake mechanism for UCNP I@PMAO@TBO in macrophages, although the contribution of other endocytic pathways, such as macropinocytosis, cannot be completely excluded.

**Figure 5:**
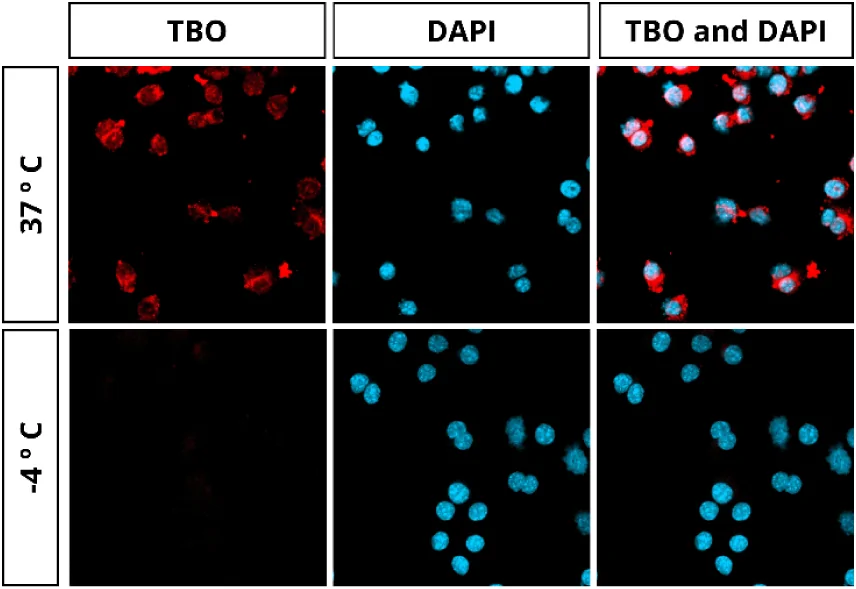
Confocal microscopy images of macrophages incubated with UCNP I@PMAO@TBO for 2 h under phagocytosis-permissive (37 °C) and phagocytosis-inhibiting (−4 °C) conditions. Images were acquired in toluidine blue O (TBO), and 4′,6-diamidino-2-phenylindole (DAPI) channels, along with merged TBO/DAPI images. While nuclear staining (DAPI) is observed in both conditions, TBO fluorescence is detected only in cells incubated at 37 °C, indicating temperature-dependent nanoparticle internalization via phagocytosis.

### 3.3 Toxicity Assay – UCNP in Cell Culture

To evaluate the toxicity of nanomaterials in cell culture media, RAW 264.7 cells were seeded in 6-well plates at a density of 8 × 10^5^ cells per well in 4 mL of RPMI-c medium and, after cell attachment, were exposed to UCNP formulations at a concentration of 100 μg mL^–1^. The cells were incubated with two different UCNP samples (UCNP I and UCNP II) for periods of 30 min, 1, 2, 4, 6, 8, 10, 12, and 24 h, with the Cell control group remaining with cells for the same period. At the end of each corresponding period, a trypan blue assay was performed, obtaining cell viability by the ratio of the number of viable cells to the number of total cells. All experiments were performed in triplicate.

What differentiates the UCNP I (17.8 nm) and UCNP II (18.7 nm) groups is the core@shell particle diameter, which implies a higher concentration of TBO per solution for larger particles, since the amount of TBO added is controlled by the area in nm^2^ of the nanoparticle surface. It was observed that there was no decrease in cell viability even after 24 h of incubation in both tested materials (Groups UCNP I and UCNP II), when compared to the Cell control group. There was also no statistically significant variation (*p* > 0.05) between the viability for the concentrations of 1 anhydride/10 amines and 1 anhydride/100 amines, as represented in the graphs in Figure 6a and 6b, which presents the data for two exposure periods, 12 and 24 h, and the cell viability of the control sample, which was kept in a culture environment until the time of the test with trypan blue. The results obtained for the other time intervals studied are available in Figure S5.

**Figure 6:**
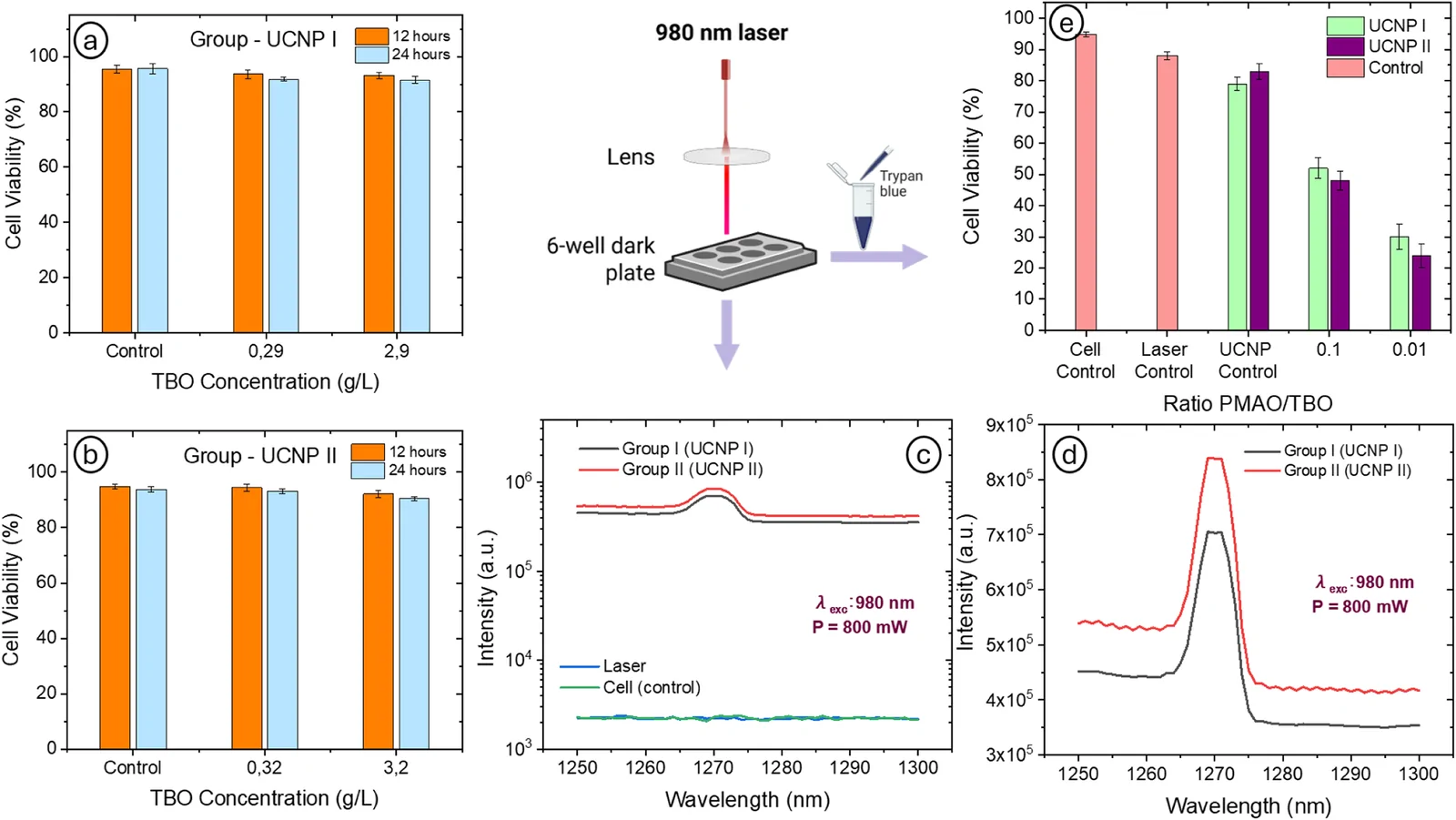
Toxicity assay and singlet oxygen detection. cell viability as a function of the concentration of photosensitizer toluidine blue O for two UCNP samples (a)­UCNP I group and (b) UCNP II group (for both (a) and (b) data are presented as mean ± standard deviation (*n* = 3). Statistical analysis was performed separately for each panel using one-way ANOVA, and no statistically significant differences were observed among the groups (*p* > 0.05). (c) Emission spectrum at 1270 nm of ^1^O_2_ from the laser blank and control cells samples with excitation at 980 nm (power, *P* = 800 mW) in comparison with the emission of the samples of group I-1 (UCNP I) and group II-1 (UCNP II); (d) emission of the samples UCNP I and UCNP II at 1270 nm with excitation at 980 nm (power, *P* = 800 mW), (e) graph of cell viability by variation of the PMAO/TBO ratio for two UCNP samples (UCNP group I and UCNP group II) after 60 s of exposure to the 980 nm laser; data are presented as mean ± standard deviation (*n* = 3). Central icon was created in BioRender. FARHAT, A. (2026) https://biorender.com/8cf2kb2.

### 3.4 Singlet Oxygen (^1^O_2_) Generation

Samples of RAW 264.7 cells were seeded at a density of 8 × 10^5^ cells per 35 mm dish in 4 mL of RPMI-c medium and incubated for 12 h in the presence of UCNP formulations at a concentration of 100 μg mL^–1^, corresponding to the experimental groups “Group I-1” – Cells + UCNP I@PMAO@TBO (TBO concentration = 1 anhydride/100 amines and 0.5% TBO + 99.5% PEG) + DAPI and “Group II-1” – Cells + UCNP II@PMAO@TBO (TBO concentration = 1 anhydride/100 amines and 0.5% TBO + 99.5% PEG) + DAPI; a control sample was also prepared and remained for the same period in a suitable environment, identified as the Cell control group. At the end of the incubation, the RPMI-c medium was removed, the well was washed with PBS (3 mL, three times), and the cells were scraped with 5 mL of PBS. They were centrifuged at 1,500 rpm for 5 min. The supernatant was discarded, and the cells were gently resuspended in 3 mL of PBS.

The Cell control group, Group I-1 (UCNP I in Figure 6c and 6d) and Group II-1 (identified as UCNP II in Figure 6c and 6d), were then stored in ice and later transferred using a pipet to a quartz cuvette suitable for luminescence analysis. For singlet oxygen detection, 800 μL of each cellular suspension was transferred to a 1 mL quartz cuvette and irradiated with a collimated 980 nm laser operating at 800 mW. The irradiation was performed for 30 s, corresponding to an irradiance of 0.8 W cm^–2^ and a total light dose of 24 J cm^–2^.

Figure 6c shows an emission spectrum at 1270 nm for the laser blank samples (signal obtained with the laser turned on at maximum power of 800 mW) and for the Cell control group, which did not present a significant signal above the equipment noise (same order of magnitude), as expected. The signals obtained for Group I-1 and Group II-1 samples are presented comparatively in Figure 6d.

It is important to emphasize that the characteristic 1270 nm emission was detected directly in suspensions containing RAW 264.7 cells previously incubated with the nanomaterials, demonstrating that singlet oxygen generation occurs under biologically relevant conditions rather than in simplified cell-free media. Despite the increased optical scattering and absorption associated with the cellular environment, both nanomaterial formulations produced detectable singlet oxygen emission, confirming the preservation of their photodynamic activity after cellular uptake and processing.

It should be noted that the TBO molecules were covalently attached to the PMAO coating through amide bonds, which are generally stable under physiological conditions and commonly employed in nanoparticle bioconjugation. Although the stability of the conjugate under phagolysosomal conditions was not directly investigated in this study, the detection of singlet oxygen after 12 h of cellular incubation indicates that a sufficient fraction of the photosensitizer remained functionally associated with the nanoparticles during the experimental period. Nevertheless, potential effects of lysosomal acidity and enzymatic activity on photosensitizer stability and aggregation warrant further investigation.

The detection of the characteristic 1270 nm emission confirmed the ability of the proposed system to generate singlet oxygen upon NIR excitation. The detection was performed after multiple washing and centrifugation steps, strongly suggesting that the observed emission originated primarily from cell-associated nanomaterials rather than from nanoparticles freely dispersed in the original culture medium. Furthermore, the direct observation of singlet oxygen phosphorescence in a cellular matrix provides strong evidence that the upconversion-mediated energy transfer process remains effective under conditions compatible with photodynamic therapy applications.

### 3.5 Toxicity Assay—UCNP + 980 nm Laser

One of central hypotheses of this study was that after excitation, the upconversion process, and energy transfer, singlet oxygen generation would occur in situ, altering cell viability and significantly inducing cell death.

Thus, after establishing that the phagocytosis process of the produced nanomaterials was possible and that the presence of UCNP inside the cell does not significantly induce cell death, it was possible to irradiate the cells with a 980 nm laser for controlled periods of time to reevaluate cell viability, considering cells that had already been incubated for 12 h in a suitable atmosphere and adhered to the culture dishes.

The results of this assay are shown in Figure 6e for samples exposed to the laser for 60 s. Cells maintained without laser exposure exhibited the highest viability and were used as the baseline control (Laser control in Figure 6e). Irradiation of cells in the absence of nanoparticles resulted in a modest decrease in viability, indicating that laser exposure alone may induce a certain degree of cellular stress (∼15%), potentially associated with local heating and other photophysical effects. A similar behavior was observed for cells incubated with nonfunctionalized UCNP I and UCNP II (UCNP control Figure 6e), demonstrating that the nanoparticles themselves do not substantially increase phototoxicity under the experimental conditions employed.

In the groups with nanoparticles, the decrease in cell viability was considerably greater, reaching close to 50% for the PMAO/TBO ratio of 0.1 and between 70 and 80% for the ratio 0.01.

In addition, direct detection of singlet oxygen emission at 1270 nm confirms the generation of ^1^O_2_ by the UCNP@PMAO@TBO system. Therefore, although a contribution from photothermal stress cannot be completely excluded, the substantially greater cytotoxicity observed for the photosensitizer-functionalized nanoparticles strongly supports a dominant contribution from photodynamically generated reactive oxygen species. The results demonstrate the ability of the synthesized nanomaterial to promote intracellular singlet oxygen generation under NIR irradiation, opening up new perspectives for the research.

## 4 Conclusion

The identification of singlet oxygen emission at 1270 nm in core@shell@PMAO@TBO samples confirmed the functionality of the synthesized material even at low concentrations in solutions containing cells with UCNP inside them, representing a significant advance in the detection of singlet oxygen in cellular media.

The application of the nanomaterial in biological samples brought new results to the field and confirmed the functionality of the proposed system, including the absorption process of UCNP emission by the photosensitizer and the energy transfer between the photosensitizer and the molecular oxygen, without the need for an additional oxygen source.

Cell viability studies indicated that the proposed nanomaterial is capable of generate singlet oxygen (corroborating the species detection results) and demonstrated its potential as a mechanism for inducing cell death. The confocal microscopy results, combined with TEM images, confirmed the UCNP’s entry into the cell via phagocytosis, opening the new possibility of targeting the material to other regions of the cell using the same process with a new modification to the material’s surface.

This new methodology has potential for application in studies of redox processes, photodynamic therapy, and imaging in complex systems, due to the high penetration of excitation sources in the biological window region (near-infrared), the photostability of NaREF_4_-based nanoparticles, and the low signal-to-noise ratio of fluorescence microscopy via upconversion.

## Supplementary Material



## Notes

Experimental details of core and core@shell UCNP synthesis and surface functionalization; TEM images and particle size distributions of UCNP II; XRD patterns confirming the hexagonal phase of UCNP I and UCNP II nanoparticles; DLS characterization of UCNP II before and after surface modification; UV–Vis absorption spectra of UCNP II and UCNP II@PMAO@TBO; emission spectra of UCNP II, UCNP II@PMAO, and UCNP II@PMAO@TBO; additional confocal microscopy images for all exposure times; additional cell viability results for all exposure times (PDF)

## #

Instituto do Coração (InCor), Department of Immunology, Hospital das Clínicas, Faculdade de Medicina, Universidade de São Paulo, Av. Dr. Enéas Carvalho de Aguiar, 44, 05403–000, São Paulo, Brasil
