# Supplementary material for: Intracellular Singlet Oxygen Generation Mediated by Upconversion Nanoparticles in RAW 264.7 Macrophages
Source: ACS Omega. 2026 Sep 4;11(37):55412–21. doi: 10.1021/acsomega.6c04049 (PMC13613876; doi:10.1021/acsomega.6c04049)
Supplement: Supplementary file 1 [file ao6c04049_si_001.pdf]

Intracellular Singlet Oxygen Generation Mediated by Upconversion Nanoparticles in RAW 264.7 Macrophages

Amanda Annunciação Farhat[1†], Bernardina Amorín Uscata[2], Magnus Ake Gidlund[3], Hiro Goto[2], Marisa Helena Gennari de Medeiros[4], Hermi Felinto de Brito[1], and Paolo Di Mascio[4*]

[1]Departamento de Química, Instituto de Química, Universidade de São Paulo, BR-05508-000 São Paulo, São Paulo, Brasil. [2]Instituto de Medicina Tropical de São Paulo, Faculdade de Medicina, Universidade de São Paulo, BR- 05403-000 São Paulo, São Paulo, Brasil. [3]Instituto de Ciências Biológicas, Universidade de São Paulo, BR-05508-000 São Paulo, São Paulo, Brasil. [4]Departamento de Bioquímica, Instituto de Química, Universidade de São Paulo, BR-05508-000 São Paulo, São Paulo, Brasil.

*corresponding author

†Present address: Instituto do Coração (InCor), Department of Immunology, Hospital das Clínicas, Faculdade de Medicina, Universidade de São Paulo, Av. Dr. Enéas Carvalho de Aguiar, 44, 05403-000, São Paulo, Brasil.

Supporting Information

S1. Preparation of UCNP@PMAO@TBO

S.1.1. Synthesis of β-$NaErF_4$: Tm^{3+} nanoparticles

To obtain the β-$NaErF_4$:Tm^{3+} core nanoparticles, a coprecipitation method based on sequential heating and cooling steps was employed using stoichiometric amounts of rare-earth acetates (1.6 mmol, 98% Er^{3+} and 2% Tm^{3+}), oleic acid (12 mL), 1-octadecene (28 mL), NH_4F (6.4 mmol), and NaOH (4.0 mmol). Initially, the rare-earth acetates, oleic acid, and 1-octadecene were introduced into a 100 mL three-neck round-bottom flask and stirred until a homogeneous solution was obtained. The reaction system was then connected to an argon line and equipped with a temperature controller.

The reaction mixture was heated to 122 °C and maintained at this temperature for 30 min, followed by cooling to room temperature. NH_4F (6.4 mmol) and NaOH (4.0 mmol), previously dissolved together in 20 mL of methanol, were simultaneously added to the reaction mixture over 30 min under constant stirring. The mixture was subsequently heated to 298 °C, maintained at this temperature for 40 min under an argon flow of 5 L min^{-1}, and finally cooled to room temperature.

S.1.2. Synthesis of β-$NaErF_4{}^{3+}$@ β-$NaYF_4$ nanoparticles

For the preparation of the β-$NaErF_4{}^{3+}$@β-$NaYF_4$ core@shell nanoparticles, the preformed core particles were employed as seeds for epitaxial shell growth (2 mL, 0.3 uM). Shell deposition was carried out through a layer-by-layer (LbL) approach adapted from previously reported methodologies, involving four sequential precursor-feeding cycles performed at 280 °C. During the isothermal growth stage, aliquots of the shell precursors (0.1 M $Y(OA)_3$ and 0.2 M Na-TFA-OA, both prepared in oleic acid) were sequentially added every 30 min under an inert argon atmosphere in an oleic acid (14 mL) and 1-octadecene (21 mL) reaction medium. The precursor stoichiometry in each cycle was adjusted according to the desired shell composition and thickness. Initially, the core nanoparticles sample, oleic acid, and 1-octadecene were placed into a 100 mL three-neck round-bottom flask and stirred until a homogeneous solution was obtained. The reaction system was then connected to an argon line and equipped with a temperature controller. The shell synthesis followed the heating sequence employed for the core preparation (25 °C → 122 °C (30 min)→ 25 °C (30 min) → 280 °C (120 min)→ 25 °C) This strategy enabled controlled shell growth while minimizing secondary nucleation events, preserving the crystalline hexagonal β-phase, and maintaining a narrow particle size distribution.

The synthetic procedure described above contains all experimental parameters necessary to identify the nanoparticle composition, reaction medium, temperature profile, and shell-growth

strategy. Certain optimization parameters related to precursor feeding and process control, which do not alter the chemical composition or general synthetic methodology, are subject to intellectual property protection under patent application BR 10 2025 025369-0 and are therefore not described in further detail.

S.1.3. Synthesis of β-$NaErF_4^{3+}$@ β-$NaYF_4$@PMAO@TBO nanoparticles

The amount of poly(maleic anhydride-alt-1-octadecene) (PMAO) was calculated based on the total surface area of the nanoparticles to achieve a surface coverage of 1.0 polymer molecule per nm^2. PMAO was dissolved in acetone (2 mL) and sonicated until complete dissolution. Solvents were evaporated under a gentle nitrogen flow, yielding dry UCNP@PMAO.

For photosensitizer conjugation, UCNP@PMAO was dispersed in borate buffer (pH 8.6), and PEG-NH_2 and TBO were added at defined molar ratios. Two formulations were prepared corresponding to 1 anhydride per 100 amines and 1 anhydride per 10 amines, maintaining a final composition of 0.5% TBO and 99.5% PEG relative to surface amine groups. The reaction was sonicated for 4 h and purified using a 100 kDa dialysis membrane. The final product was recovered in HEPES buffer (50 mM, pH 7.5).

S2. Experimental Groups

Cells were seeded on culture plates or glass coverslips, depending on the assay, and allowed to adhere for 1 h prior to nanoparticle exposure.

Two UCNP core@shell systems with identical composition (β-$_{0.98}NaErF_4{:}_{0.02}Tm^{3+}$@$NaYF_4$) and distinct average diameters were evaluated: UCNP I (17.8 nm) and UCNP II (18.7 nm), both at a concentration of 100 μg mL^{-1}.

For UCNP@PMAO@TBO samples, two photosensitizer loadings were investigated (1:100 and 1:10 anhydride-to-amine ratios). DAPI was used for nuclear staining at a final concentration of 1 μg mL^{-1}.

Experimental groups included:

- Cell Control – Only RAW Macrophages cells
- DAPI control – RAW Macrophages + DAPI
- UCNP I control – RAW Macrophages + UCNP I (without PMAO and TBO)
- UCNP II control - RAW Macrophages + UCNP II (without PMAO and TBO)
- Group I-1 : UCNP I@PMAO@TBO (1:100)
- Group I-2 : UCNP I@PMAO@TBO (1:10)
- Group II-1 : UCNP II@PMAO@TBO (1:100)
- Group II-2 : UCNP II@PMAO@TBO (1:10)

S3. Instrumentation and Characterization Methods

S3.1. X-ray Diffraction (XRD)

XRD patterns were recorded using Rigaku Miniflex II operating with Cu Kα radiation (λ = 1.5406 Å) at 30kV and 15mA. Samples were prepared by drop-casting dried UCNP powders onto silicon substrates. Diffraction patterns were collected over a 2θ range of 10–70° with a step size of 0.1° and a counting time of 5s per step.

S3.2. Transmission Electron Microscopy (TEM)

TEM images of UCNPs and cell sections were acquired using a JEOL 2100 operating at an accelerating voltage of 100 kV and 200 kV. For nanoparticle analysis, samples were dispersed in cyclohexane and deposited onto carbon-coated copper grids. For cellular TEM, RAW 264.7 cells were fixed, dehydrated, embedded in resin, sectioned into ultrathin slices, and stained according to standard protocols prior to imaging.

TEM samples were prepared by diluting the nanoparticle suspension in cyclohexane (1:100, v/v). Subsequently, 5 µL of the diluted suspension was deposited onto a carbon-coated copper grid and allowed to dry under ambient conditions for at least 24 h before imaging.

S3.3. Dynamic Light Scattering (DLS)

Hydrodynamic diameter distributions was measured using a Zetasizer Nano ZS90 instrument (Malvern Panalytical). Measurements were performed in quartz cuvettes. The instrument operates over a particle size range of approximately 0.3 nm to 3 µm.

S3.4. UV–Vis Absorption Spectroscopy

UV–Vis absorption spectra were recorded using a Shimadzu UV-2600 spectrophotometer. Measurements were performed using quartz cuvettes with optical volumes of 1 mL and 3 mL, depending on sample availability and concentration.

S3.5. Fluorescence and Upconversion Emission Spectroscopy

Upconversion emission spectra were recorded under continuous-wave 980 nm laser excitation with a maximum power of 800-1600 mW. Emission was collected using a Horiba-PTI Fluorolog-QM, covering the visible and near-infrared ranges.

The photophysical study of singlet oxygen monomolecular emission at 1270 nm in dispersions/solutions containing nanoparticles and photosensitizers was performed on a spectrofluorometer (Edinburgh Analytical Instruments – UK) coupled with a photomultiplier tube, consisting of a photomultiplier tube (R5509 – Hamamatsu Photoniks KK – Shizuoka, Japan) sensitive to the infrared region (800-1400 nm), cooled to -80 ºC by a liquid N2 cooling system (S600 PHOTOCOOLTM, PC176TSCE005 cooler – Products for Research Inc. – MA, USA).

S3.6. Confocal Laser Scanning Microscopy (CLSM)

Confocal microscopy images were obtained using a Zeiss Confocal Microscopy LSM 800 equipped with 40× and 63× objectives. DAPI was excited at 465 nm and detected in the 410–590 nm range, while toluidine blue O (TBO) fluorescence was excited using the Alexa Fluor 610 channel (612 nm) and detected in the corresponding emission window (585-700 nm). Image acquisition parameters were kept constant across all samples to allow qualitative comparison.

S4. Results

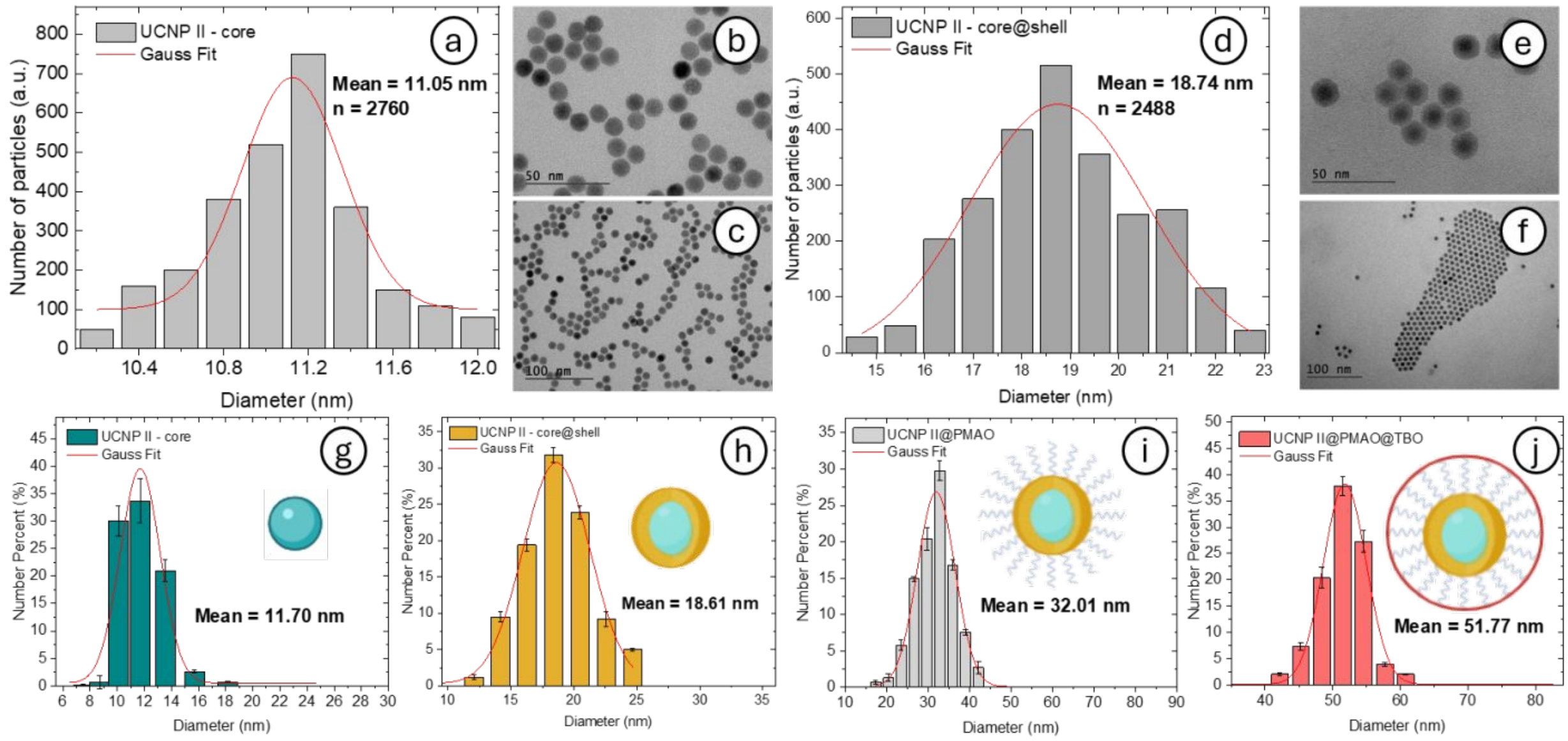

Figure S1. Structural and colloidal characterization of UCNP II. (a) Size-distribution histogram of β-$NaErF_4^{3+}$ core nanoparticles obtained from TEM analysis (n = 2760 particles). (b,c) Representative TEM micrographs of the core nanoparticles. (d) Size-distribution histogram of β-$NaErF_4^{3+}$@β-$NaYF_4$ core@shell nanoparticles obtained from TEM analysis (n = 2488 particles). (e,f) Representative TEM micrographs of the core@shell nanoparticles. Hydrodynamic diameter distribution obtained by dynamic light scattering (DLS) for (g) UCNP II core nanoparticles. (h) UCNP II core@shell nanoparticles. (i) UCNP II@PMAO nanoparticles after surface modification. (j) UCNP II@PMAO@TBO nanoparticles after photosensitizer conjugation. The progressive increase in hydrodynamic diameter after shell growth and surface functionalization is consistent with successful nanoparticle modification.

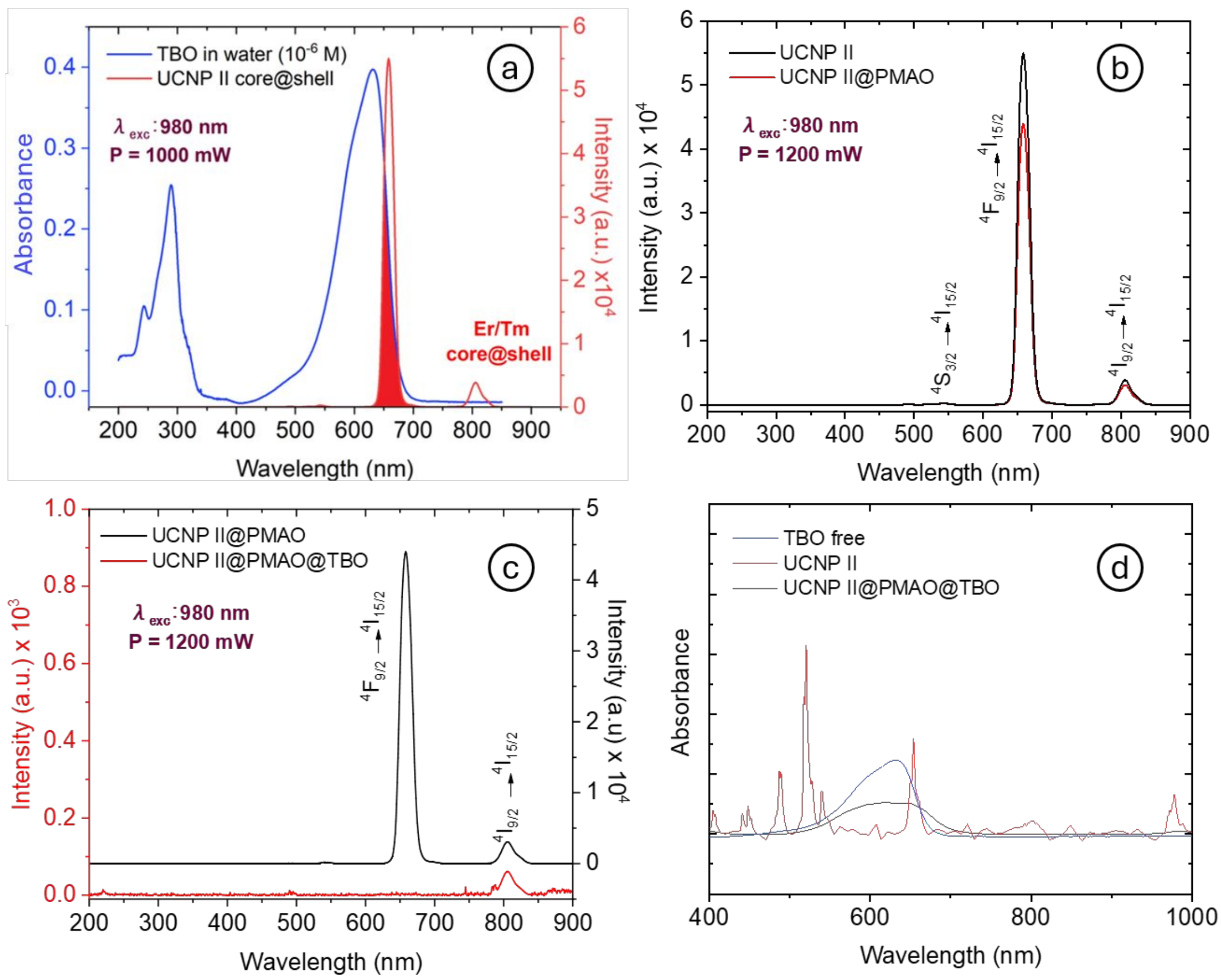

Figure S2. Optical characterization of UCNP II before and after surface functionalization. (a) Spectral overlap between the emission spectrum of β-$NaErF_4^{3+}$@β-$NaYF_4$ nanoparticles dispersed in cyclohexane (λexc = 980 nm, P =

1000 mW) and the absorption spectrum of toluidine blue O (TBO) in water, demonstrating overlap in the 500–700 nm region. (b) Emission spectra of core@shell and core@shell@PMAO nanoparticles recorded in cyclohexane (λexc = 980 nm, P = 1200 mW). (c) Emission spectra of core@shell@PMAO nanoparticles recorded in water before and after TBO conjugation (λexc = 980 nm, P = 1200 mW), showing suppression of the 658 nm emission band. (d) UV–Vis absorption spectra of free TBO, core@shell nanopar-ticles, and UCNP@PMAO@TBO.

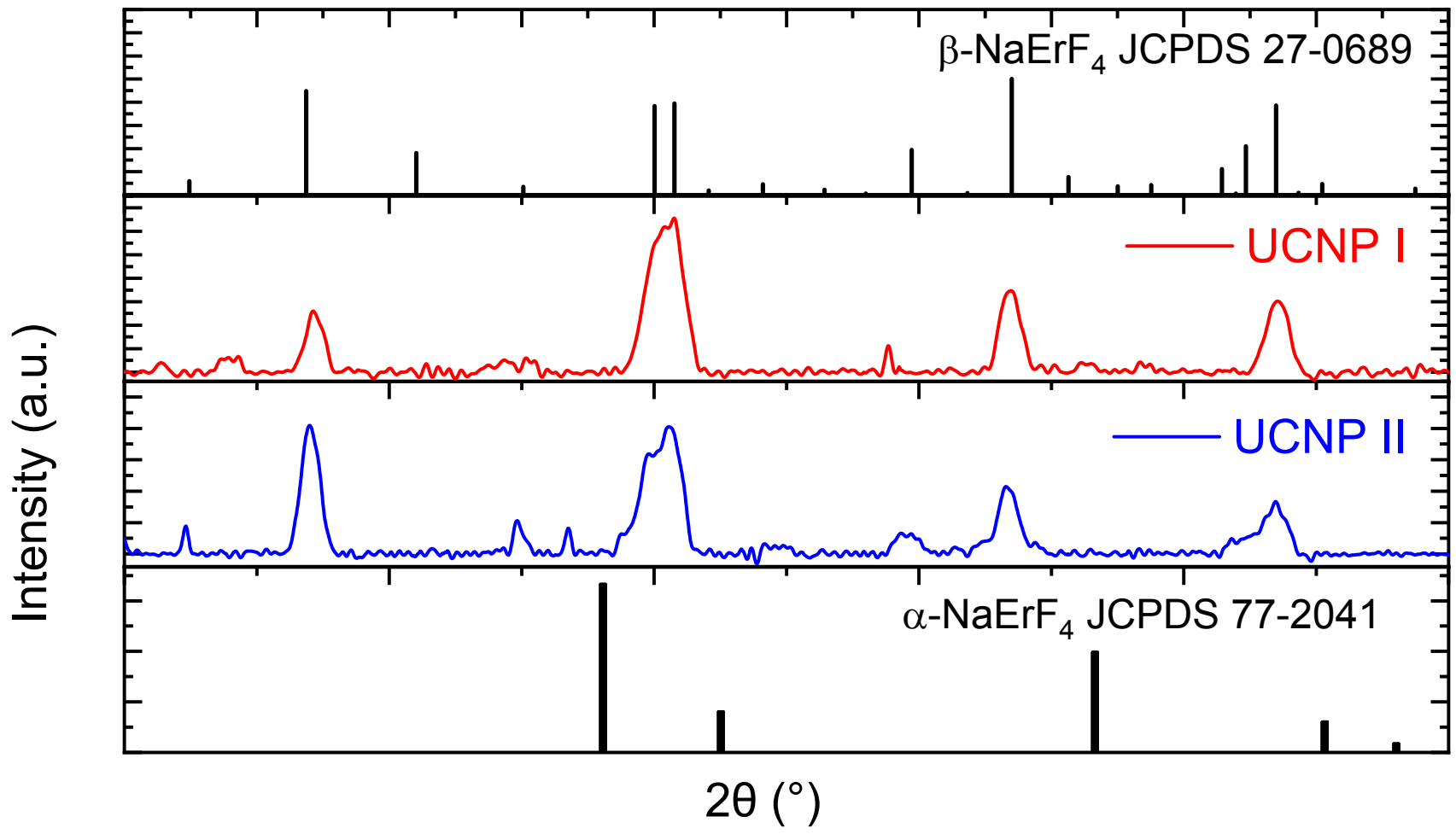

Figure S3. Structural characterization of β-$Na_{0.98}ErF_4$:$_{0.02}Tm^{3+}$ UCNP I and UCNP II upconversion nanoparticles. XRD patterns of the as-synthesized samples compared with the standard diffraction patterns of α-$NaErF_4$ (JCPDS 77-2041) and β-$NaErF_4$ (JCPDS 27-0689)

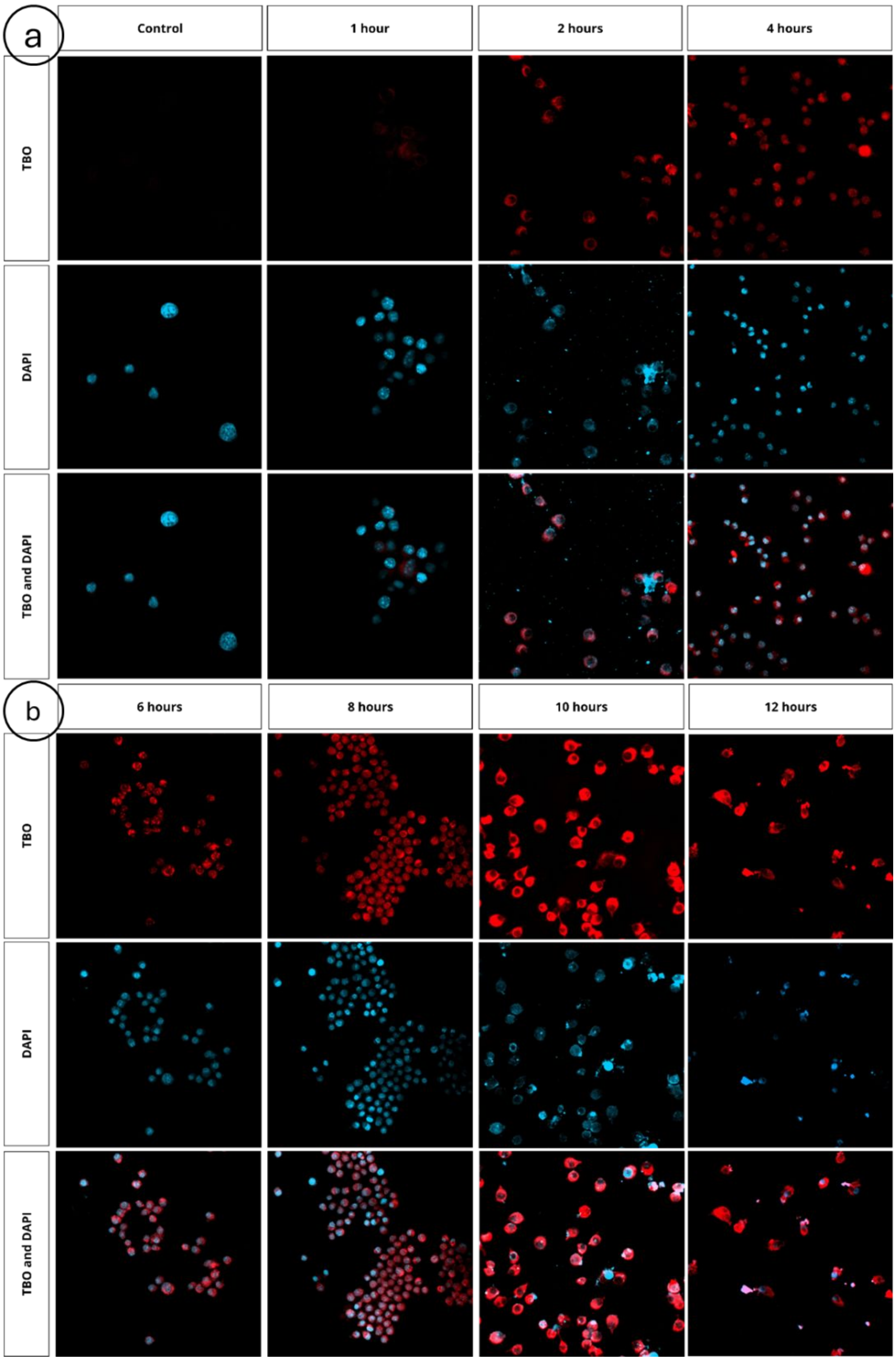

Figure S4. Images obtained by confocal microscopy (CO) for Toluidine Blue O (TBO), and 4′,6′-diamino-2-phenylindole (DAPI) channels and overlay of the DAPI and TBO channels for (a) 1, 2, and 4 hours of cell culture exposure to UCNPs and control and (b) for 6, 8, 10, and 12 hours of cell culture exposure to UCNPs.

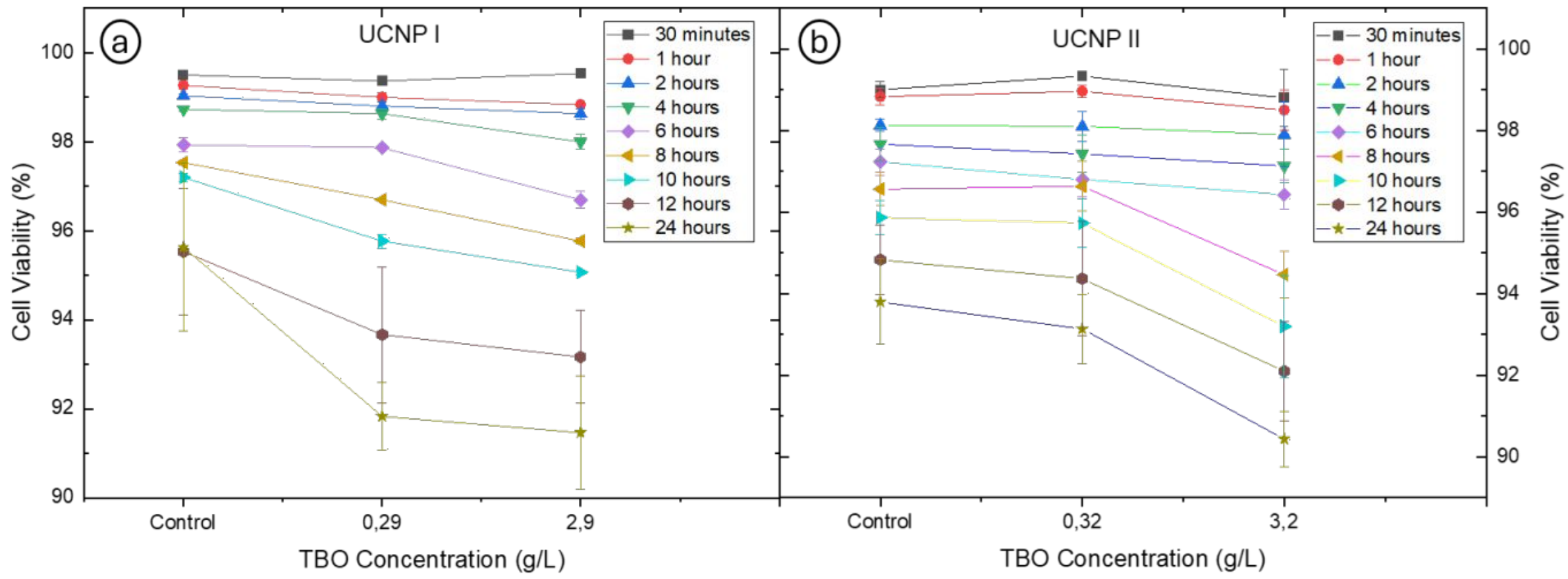

Figure S5. Graph of cell viability versus variation in the concentration of photosensitizer (PS) toluidine blue O (TBO) over periods of 30 minutes, 1, 2, 4, 6, 8, 10, 12, and 24 hours for (a) UCNP I@PMAO@PS sample and (b) UCNP II@PMAO@PS.
